# The effects of cannabidiol on impulsivity and memory during abstinence in cigarette dependent smokers

**DOI:** 10.1038/s41598-018-25846-2

**Published:** 2018-05-15

**Authors:** C. Hindocha, T. P. Freeman, M. Grabski, H. Crudgington, A. C. Davies, J. B. Stroud, R. K. Das, W. Lawn, C. J. A. Morgan, H. V. Curran

**Affiliations:** 1 Clinical Psychopharmacology Unit, University College London, WC1E 7HB London, United Kingdom; 2 National Addiction Centre, Institute of Psychiatry, Psychology and Neuroscience, King’s College London, London, SE5 8BB United Kingdom; 3 School of Experimental Psychology, University of Bristol, 12a Priory Road, BS81TU Bristol, United Kingdom; 4 Psychopharmacology and Addiction Research Centre, University of Exeter, Devon, UK

## Abstract

Acute nicotine abstinence in cigarette smokers results in deficits in performance on specific cognitive processes, including working memory and impulsivity which are important in relapse. Cannabidiol (CBD), the non-intoxicating cannabinoid found in cannabis, has shown pro-cognitive effects and preliminary evidence has indicated it can reduce the number of cigarettes smoked in dependent smokers. However, the effects of CBD on cognition have never been tested during acute nicotine withdrawal. The present study therefore aimed to investigate if CBD can improve memory and reduce impulsivity during acute tobacco abstinence. Thirty, non-treatment seeking, dependent, cigarette smokers attended two laboratory-based sessions after overnight abstinence, in which they received either 800 mg oral CBD or placebo (PBO), in a randomised order. Abstinence was biologically verified. Participants were assessed on go/no-go, delay discounting, prose recall and N-back (0-back, 1-back, 2-back) tasks. The effects of CBD on delay discounting, prose recall and the N-back (correct responses, maintenance or manipulation) were null, confirmed by a Bayesian analysis, which found evidence for the null hypothesis. Contrary to our predictions, CBD increased commission errors on the go/no-go task. In conclusion, a single 800 mg dose of CBD does not improve verbal or spatial working memory, or impulsivity during tobacco abstinence.

## Introduction

Nicotine withdrawal consists of multiple physiological, affective and cognitive symptoms that can peak within hours of stopping smoking^1–3^. Grabski, *et al*.^4^ recently conducted a meta-analysis of cognitive tasks sensitive to tobacco abstinence. Abstinent smokers, in comparison to satiated smokers, show greater impulsivity on two specific tasks, *delay discounting* (defined as the degree to which one prefers smaller, immediate rewards over larger, more delayed rewards^5^) and *response inhibition* (the ability to stop a pre-potent response e.g. craving for cigarettes; a marker of executive functioning; and theoretically important for successful smoking cessation^6^). Abstinent smokers also showed impaired arithmetic and recognition memory ability, both of which includes a core component of working memory and were therefore interpreted as potential evidence for effects of abstinence on working memory^7,8^. Therefore, pharmacotherapies targeting improving cognition during tobacco abstinence may be useful for the treatment of tobacco use disorders.

Cannabidiol (CBD), the non-intoxicating cannabinoid found in cannabis, may have a novel application in nicotine withdrawal^9^. Thus far, CBD has been shown to reduce craving in both pre-clinical and clinical models of heroin addiction^10,11^. Furthermore, it may have a specific utility in cigarette smoking. Morgan, *et al*.^12^ found that a single week of ad-hoc CBD via inhaler, compared to placebo, reduced cigarette smoking by almost 40%, however craving was unaffected. Hindocha *et al*.^9^ found that 800 mg oral CBD, in comparison to placebo, reversed attentional bias away cigarette cues, and reduced explicit liking of cigarette stimuli but this also occured in the absence of changes in withdrawal and craving. CBD may also have pro-cognitive effects and has, in multiple studies, been shown to protect against the detrimental cognitive effects of THC, and particularly in the domains of verbal episodic and recognition memory^13–16^. In regards to impulsivity, Bhattacharyya, *et al*.^17^ found opposite effects of THC and CBD in the para-hippocampal gyrus during a response inhibition task. Borgwardt, *et al*.^18^ found CBD deactivated the left temporal cortex and insula but was not associated with increases in regional activity relative to placebo. Finally, no research has investigated the effects of CBD on delayed discounting.

Experimental medicine approaches to study tobacco abstinence are cost-effective and mechanistic evaluations of a medication, and may facilitate drug discovery^19^. We hypothesise that after overnight cigarette abstinence in dependent cigarettes smokers, CBD would improve performance in working and verbal episodic memory and on impulsivity tasks, in comparison to placebo.

## Results

### Demographics and Manipulation Checks

Participants (n = 30; 15 female) were 28.07 (±8.66) years old with an FTND score of 5.56 (±1.13) which is considered moderate-to-high cigarette dependence. Participants smoked 13.5 (±2.39) cigarettes per day for the past 9.55 (±7.36) years. They had been smoking 10 + cigarettes per day for the past 8.17 (±7.08) years. They smoked their first cigarette 25.50 (±15.87) minutes after waking. Drug use in this population was minimal (see Table 1). Barratt Impulsiveness Scale (BIS-11) score was 75.17 (±5.31). Trait anxiety as measured by the State Trait Anxiety Inventory-Trait (STAI) was 40.93 (9.40). Carbon monoxide (CO) upon arrival was 4.27 ppm (±2.23) for CBD and 4.17 (±2.69) for PBO (t_(29)_ = 0.324, *p* = 0.748). Withdrawal, as measured by the Mood and Physical Symptoms Scale (MPSS) upon arrival was significantly greater for CBD (12.03 ± 3.13) and PBO (12.13 ± 3.72) sessions in comparison to satiation (9.97 ± 2.86; both *p’s* < 0.05).

**Table 1:** Drug use history (N = the number of people who used the drug in the past year).

N	ALCOHOL	CANNABIS	MDMA	COCAINE
	26	17	9	9
LAST USED (DAYS)	6.39 (10.13)	100.00 (68.30)	84.66 (82.22)	100.00 (56.12)
YEARS USED	13.08 (8.68)	8.29 (4.61)	4.55 (1.59)	3.33 (2.12)
DAYS PER MONTH	11.43 (8.85)	0.75 (1.30)	0.67 (1.32)	0.5 (1.15)
AMOUNT PER SESSION	7.10 units (3.23)	0.87 joints (0.69)	258.33 mg (144.70)	800 mg (0.83)

Means (Standard Deviation) are presented.

### Impulsivity

#### Delay discounting

There was no main effect of drug (*F*_(1,29)_ = 0.065, *p* = 0.801, *ηp*^2^ = 0.002) suggesting no difference between CBD (M: 0.006, SE:0.001) and PBO (M: 0.006, SE:0.001). This was confirmed by Bayesian analysis which showed that the null was 4.61 times more likely than the alternative given the data (JZS Bayes Factor: 4.61).

#### Go/no-go

There was a main effect of drug (F_(1,29)_ = 4.721, *p* = 0.038, *ηp*^2^ = 0.140) which showed there were more commission errors after CBD (M: 2.600, SE:0.400) compared to PBO (M: 1.900, SE: 0.350).

### Memory

#### Prose recall

There was no main effect of drug (*F*_(1,29)_ = 1.410, *p* = 0.244, *ηp*^2^ = 0.046) suggesting no effect of CBD (M: 8.790, SE: 0.690) in comparison to PBO (M: 9.740, SE: 0.590). This was confirmed by a Bayesian analysis that indicated the null was 3.61 times more likely than the alternative given the data, providing evidence that CBD did not affect verbal memory (JZS Bayes Factor= 3.61). However, there was main effect of delay (*F*_(1,29)_ = 57.020, *p* < 0.001, *ηp*^2^ = 0.660) which showed delayed recall (M: 8.283, SE:0.574) was poorer than immediate recall (M: 9.272, SE:0.574). There was no interaction between condition and delay (*F*_(2,58)_ = 0.530, *p* = 0.471, *ηp*^2^ = 0.018).

#### N back

Correct responses: There was no main effect of drug (*F*_(1,29)_ = 0.532, *p* = 0.472, *ηp*^2^ = 0.018) suggesting no effect of CBD (M: 42.87, SE: 0.61) in comparison to PBO (M: 43.21, SE: 0.58). The lack of main effect of drug was confirmed by a Bayesian analysis which showed that null was 5.48 times more likely than the alternative hypothesis given the data (JZS Bayes Factor = 5.48). There was also a main effect of load (*F*_(1,32)_ = 53.022, *p* < 0.001 *ηp*^2^ = 0.646) which showed that correct responses decreased as a function of load (0-back M: 47.63 SE: 0.19, 1-back M: 43.32 SE: 0.48, 2-back M: 38.17 SE:1.27). There was no drug x load interaction (F_(2,58)_ = 1.776, *p* = 0.178, *ηp*^2^ = 0.058).

Reaction time: There was no main effect of drug suggesting no difference between CBD (M: 527.93, SE: 18.85) and PBO (M: 531.84, SE: 14.52). This was confirmed by a Bayesian analysis which showed that the null was 6.66 more likely than the alternative (JZS Bayes Factor: 6.66) There was a main effect of load (*F*_(1,41)_ = 96.811, *p* < 0.001, *ηp*^2^ = 0.769) which showed that RT increased with load (0-back M: 412.57 SE: 12.63, 1-back M: 536.61 SE:16.86, 2-back M: 640.47 SE:24.11). No interactions emerged.

Maintenance and Manipulation: There was no main effect of drug for maintenance (*F*_(1,29)_ = 0.118, *p* = 0.734, *ηp*^2^ = 0.004), suggesting no difference between CBD (M: −3.73, SE: 0.56) and PBO (M: −4.03, SE: 0.67). There was no main effect of drug for manipulation (*F*_(1,29)_ = 3.047, *p* = 0.091, *ηp*^2^ = 0.095) suggesting no difference between CBD (M: −6.73, SE: 1.39) and PBO (M: −4.40, SE: 1.36).

### Order effects

**Figure 1:**
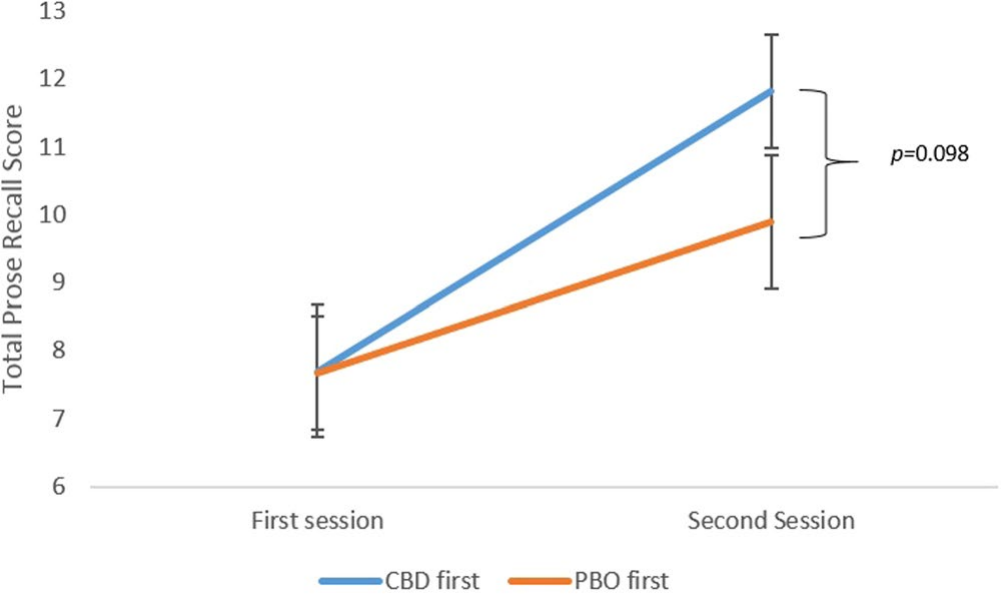
Order effects for the prose recall task. Error bars represent ± SEM.

Order effects emerged for the prose recall task. A drug x order interaction emerged when order was included as a between subjects factor (F(1,28) = 33.037, p < 0.001, *ηp*^2^ = 0.540) which showed that the prose recall score was dependent upon which drug was received in the first session. To follow this up a session (one, two) x drug order (CBD first, PBO first) mixed ANOVA was conducted which showed a session x order interaction (F(1,28) = 5.032, p = 0.033, *ηp*^2^ = 0.015). This revealed a trend towards a difference between orders for session two (*p* = 0.098) wherein the participants who received CBD first improved to a greater extent on the second session than those who received placebo first (Fig. 1). For session one, they were equivalent (*p* = 0.978). Additionally, practise effects for observed for both orders but those who received CBD first increased by 4.12 (SE: 0.75) idea units between session one and two (*p* < 0.001), and those who received PBO first increased by 1.75 (SE: 0.75) idea units between session one and session two (*p* = 0.026). No order effects emerged for the remainder of the tasks.

## Discussion

This study aimed to investigate if CBD, in comparison to placebo, would improve memory and reduce impulsivity in dependent smokers during tobacco abstinence. We selected tasks and domains that have been shown to be impaired during cigarette abstinence in a recent meta-analysis^4^. There were no effects of CBD on prose recall, spatial working memory (correct responses, reaction time, maintenance and manipulation), or delay discounting tasks. We obtained evidence in support of the null for these comparisons using Bayesian analyses. Contrary to our predictions, however, CBD increased commission errors compared to placebo on the go/no-go task. Finally, we observed order effects on the prose recall task which suggest that those who were randomised to be given CBD first, showed slightly greater improvement between session one and two, than those given placebo first, tentatively supporting the pro-cognitive effects of CBD.

Impaired response inhibition is an important etiological factor in tobacco dependence^20–22^. Response inhibition may be a key cognitive process during tobacco withdrawal as it requires inhibiting a pre-potent response e.g. automatically picking up a cigarette and/or inhibiting the urge to smoke. However, there were no beneficial effects of CBD on the number of commission errors. Indeed, we show here that CBD actually increased commission errors. This is an unexpected finding from a single study and therefore should be should be interpreted as preliminary evidence until it is replicated. Furthermore, this study did not find that CBD modified responses on delay discounting.

Grabski, *et al*.^4^ also showed impaired arithmetic and recognition memory ability, in abstinent smokers, interpreted by the authors as potential evidence for effects of abstinence on working memory. However, recognition memory also includes a component of verbal episodic memory. In the present study, there was no difference between CBD and placebo on either verbal episodic and working memory. Previous research has suggested that CBD may protect against THC-induced impairments in verbal/recognition memory^13–15^. Order effects were observed between the two abstinent sessions for the prose recall task where if participants were given CBD in the first session, then they performed better in the second session. However, if participants were given placebo in the first session, then they still improved (as a function of practise) however the improvement was not as great as with CBD. These effects were found despite attempts to minimise practise effects between the two abstinent sessions.

The generally null results of CBD on cognition here may not be surprising as the mechanisms responsible for the effects of CBD on cognition are poorly understood. The effects of CBD are not consistent for even its most well studied constructs such as lessening of acute anxiety^23^. They likely are dependent on experimental setting, dose, dosing regimen, route of administration, the population studied and whether CBD is given in combination with THC.

The strongest evidence for the utility of CBD within addiction may arise from those tasks specifically associated with the motivational salience of cues associated with drug use^9–11,24^. For example, Ren *et al*.^10^ conducted a preclinical study investigating heroin self-administration and found that although self-administration itself was unaffected by CBD, cue-induced heroin-seeking behaviour and reinstatement were both reduced. CBD also inhibited relapse behaviour during active heroin intake. In regards to human research, Hurd, *et al*.^11^ conducted a pilot double-blind, placebo-controlled investigation in opioid-dependent individuals who were abstinent for 7 days. They found that cue-induced craving was significantly reduced after a single administration of CBD, and this persisted for 7 days. In regards to the effects of CBD on cigarette smoking, Morgan, *et al*.^12^ found a 40% reduction in cigarettes smoked after one week of ad-lib CBD inhaler vs. placebo, however no mechanisms were investigated. The study was based on previous research showing that higher levels of CBD in smoked cannabis reduced the “wanting” and “liking” of cannabis related stimuli^24^. Finally Hindocha *et al*.^9^ found that CBD reversed attentional bias away from cigarette cues, compared to placebo, in abstinent, dependent cigarette smokers. Participant’s attentional bias under CBD was therefore no longer different from satiety. Moreover, Hindocha *et al*. found a reduction in explicit “pleasantness” of cigarette cues. Taken together, these findings are consistent with the possibility that CBD has utility in modification of the salience of drug cues (bottom-up cognition) and not necessarily with the modulation of top-down cognitive processes such as impulsivity. Further investigation is required to confirm this.

This study has several methodological strengths including using tasks and domains that have been previously been shown to be impaired during tobacco abstinence^4^. Furthermore, this study had a moderately large sample size in a crossover design, informed by a power calculation and inflated by 50% to account for “winner’s curse”. The experimental medicine design of the study allowed for an economical and mechanistic evaluation of CBD on tobacco withdrawal. Finally, abstinence was confirmed by biological verification (carbon monoxide). However, there are also some limitations. Given the incompletely elucidated mechanism of CBD, the present study may have not selected the correct dose for the therapeutic effects of CBD. There has only been one published dose-response study of CBD in humans which was specifically designed to test the anxiolytic effects in public speaking but only tested three doses^25^. Therefore dose selection generally copies that used in previous single-dose studies. The dose-response effects of CBD may follow an inverted U shaped curve, and thus our 800 mg dose may be too high for the therapeutic dose window^25^. We did not collect plasma to monitor the pharmacokinetics of CBD. However, this study was informed by previous pharmacokinetic data from the same 800 mg oral dose of CBD^26^. Furthermore, only a single dose of the drug was given, and it may be that CBD is more effective with repeated dosing^25^. Future research should investigate multiple doses and repeated administration to reach plasma concentrations that are at a steady-state. Finally, some participants in our sample (17 out of 30) had a history of cannabis use although none were current cannabis users (confirmed by urinalysis and drug history). The average last use of cannabis was 100 days previously (over 3 months; See Table 1). It may be that the effects of CBD are dependent on cannabis use history, therefore future hypothesis-driven studies should investigate if CBD has differential effects in cannabis users and cannabis naïve volunteers.

In conclusion, this study finds that in dependent cigarette smokers who were abstinent overnight, CBD did not improve cognition on tasks that have been shown to be impaired during cigarette abstinence and this was confirmed by Bayesian analyses in support of the null hypothesis. This research suggests that CBD is not efficacious in reversing the cognitive impairments associated with acute nicotine abstinence in cigarette dependence, although future research is required to investigate different doses and dosing schedules.

## Methods

### Design and participants

Thirty participants attended 3 sessions (7.85 ± 2.77 days between sessions). They smoked as normal before their first (baseline) session, verified with expired Carbon Monoxide (CO) ≥10 parts per million (ppm) (Bedfont Scientific, Harrietsham, UK). Participants then attended two sessions after overnight (mean: 11 hours, range: 9.5–13 hours) abstinence, verified by CO ≤ 10 ppm^27^. A double-blind placebo-controlled crossover design was implemented to compare the effects of 800 mg oral CBD with matched placebo (PBO) after overnight abstinence. Treatment order for abstinent sessions was randomised and counterbalanced. Additional data from this study has been reported elsewhere^9^.

Dependent cigarette smokers were recruited from the local community and through online message boards. Inclusion criteria were: (i) age 18–50 years; (ii) smoking ≥10 cigarettes a day for at least the last year; (iii) Fagerström Test of Nicotine Dependence (FTND) score ≥4 (moderate dependence)^28^; (iv) smoking first cigarette within an hour of waking; (iv) negative instant drug urine screen for all drugs of abuse on the first session. Exclusion criteria were: (i) use of nicotine replacement therapy or any other nicotine pharmacotherapy; (ii) self-reported recent (past 4 weeks) use of cannabis or other illicit drugs; (iii) recent (past 4 weeks) or ongoing use of e-cigarettes; (iv) current, self-reported mental health, physical health or learning impairments; (v) pregnancy or breast feeding; (vi) allergies to cannabidiol, gelatine, lactose, microcrystalline cellulose or chocolate.

### Power calculation

We calculated an N of 20 would be necessary to have power of 0.95 to detect a large effect size of d = 0.78 (F = 0.38). This was based on the difference in the number of cigarettes smoked pre- to post- one week of CBD inhaler vs. placebo in Morgan *et al*.^12^. This sample size was increased by 50% to adjust for “winner’s curse”, or the tendency for effect sizes estimates from an initial positive finding to be over-inflated^29^ yielding a final sample of 30.

### Drug administration

Participants were administered 800 mg oral CBD doses (pure synthetic (−)-CBD, STI Pharmaceuticals, Essex, England) or matched placebo (lactose powder) in matched capsules in a double-blind, counterbalanced manner. The 800 mg dose was chosen as it has shown clinical efficacy for schizophrenia^30^. 800 mg CBD is well tolerated, shows no abuse liability, does not modify the reinforcing properties of smoked cannabis^26,31^ or exacerbate the adverse effects of fentanyl^32^. 800 mg per day for three days has been shown to reduce anxiety and cue induced craving in individuals addicted to opiates who had been abstinent for a week^11^. 600 mg has been shown to influence neural networks that include medial temporal, prefrontal and striatum brain regions therefore 800 mg should have a similar effect^33^. 800 mg produces an increase in plasma concentrations after administration (Cmax = 77.9 ± 25 ng/mL, Tmax = 180 minutes)^26,31^. The oral route of administration was chosen in comparison to inhaled because data on plasma concentrations was available. Furthermore, there is far higher levels of variability with the inhaled route which is dependent on how much is exhaled, breath holding protocols and bioavailability which is not yet reported^34^. Finally, CBD, when vaporised can be irritating for the throat which generates a cough^34^.

### Assessments

#### Impulsivity

Delay discounting task^35^: In this task, participants had to make 91 alternative forced choices between a standard hypothetical amount of money (£100) available after one of five delays (0, 7, 30, 90, or 180 days) and one of 23 alternative hypothetical amounts available immediately (e.g. “Which would you prefer: £100 in 180 days or £30 now?”). The indifference parameter (k), which was the main variable of interest was derived from the indifference points from each session and calculated according to Reed, *et al*.^36^.

Go/no-go task^37^: This task required participants to make a response when a designated “go” cue (Star) was presented and withhold responding to a designated “no-go” cue (Arrow). Each trial began with a fixation cross displayed for 500 ms. The cues were shapes presented in the center of a screen for 1000 ms. A practice phase of 6 trials was implemented, where participants received feedback on their performance. The first 20 trials were go-trials to build a pre-potent response and the remaining 90 trials were made up of 30 no-go trials and 60 go-trials, presented in randomized order. The main variable of interest was the commission errors (i.e. going on NoGo trials).

#### Memory

Prose recall: The Prose Recall subtest of the Rivermead Behavioral Memory Test^38^ taps episodic memory. Participants heard a 30 s passage of prose (a news bulletin) and recalled its contents immediately and after a delay of 25 minutes. The primary outcome is the mean number of idea units recalled. Three versions were presented in a counterbalanced order across each of the three sessions.

N back: This task assesses spatial working memory. Visual stimuli appeared in one of six different locations around a central fixation cross on the computer screen, in a sequential order^39,40^. Participants responded by pressing a “Yes” or “No” key according to whether a) the stimuli appeared in a pre-defined location (0-back; attentional control), b) whether the stimulus was in the same position as the stimulus one before (1-back), and subsequently, (c) two before (2-back). Reaction time and Accuracy were recorded.

#### Procedure

Following telephone screening, potentially eligible participants attended a baseline session to provide informed consent, confirm their smoking status, provide a urine sample to confirm no recent recreational drug use, take a pregnancy test (if female), receive instructions for abstinent sessions and complete the task battery. The satiated session occurred first in a fixed order to minimize practice effects between the following two sessions, optimizing the design for comparing CBD and placebo during abstinence (this data has been published in Hindocha, *et al*.^9^). Participants were instructed to remain abstinent from midnight the night before the two experimental ‘abstinent’ sessions resulting in an average of 11 hours abstinence (range 9.5–13 hours). Each abstinent session began with confirmation of cigarette abstinence (breath CO) and assessment of craving (measured by the Questionnaire of Smoking Urges-Brief^41^ and withdrawal (measured by the Mood and Physical Symptoms Scale^42^), these data can be found in Hindocha, *et al*.^9^. Next, drug administration took place. Trait questionnaires were conducted immediately after this and were equally split between the two abstinent sessions. Testing began 150 minutes after drug administration so CBD would reach peak levels and occurred in the following order: prose recall immediate, N back (0-back, 1-back, 2-back), delay discounting, Go/No-Go, prose recall delayed. Smoking was not permitted until the end of the session. All participants provided written informed consent. Ethical approval was given by UCL Ethics Committee. Participants were reimbursed £10/hour. The experiment was conducted in accordance with the Declaration of Helsinki and all data was processed and stored according to the Data Protection Act 1998.

## Statistical Analysis

Statistical analyses were performed in the Statistical Package for Social Scientists (SPSS 23; IBM, Chicago, IL). Visual inspection of diagnostic plots was used to check for normality. Where sphericity was violated, the Greenhouse Geiser correction was used and degrees of freedom were rounded to the nearest integer. Outliers > 1.5 x interquartile range (IQR) were winsorized to the next highest/lowest value. Logged-k values (delay discounting) were used as the data showed a non-normal distribution. We conducted paired sample t-tests between both drug conditions and satiety, to confirm that abstinence increased withdrawal^9^. The prose recall, N back, Delay Discounting and Go/No-Go were analysed using repeated measures ANOVA with a factor of drug (CBD, PBO) and additional task specific factors for the prose recall (immediate, delayed) and for the N back (0 back, 1 back, 2 back). Interactions were explored via pairwise post-hoc comparisons, Bonferroni-corrected locally within each omnibus term to avoid an inflated Type I error rate. Order effects were analysed with drug order (CBD first, PBO first) as a between subjects factor. As we did not have any specific *a priori* hypotheses regarding covariates, we did not include any as per Kraemer^43^.

Scaled Jeffreys-Zellner-Siow (JZS) Bayes Factor was calculated for the main effect of drug (CBD vs. PBO) when it was not significant according to frequentist statistics^44,45^. This was calculated for the main variable of interest in each task (k, prose recall total, N back total correct responses). We used a scaled-information prior of r = 1, recommended by Rouder, *et al*.^46^ as default.

The dataset generated during the current study are available from the corresponding author on reasonable request.
